# Staging of Skin Cancer Based on Hyperspectral Microscopic Imaging and Machine Learning

**DOI:** 10.3390/bios12100790

**Published:** 2022-09-25

**Authors:** Lixin Liu, Meijie Qi, Yanru Li, Yujie Liu, Xing Liu, Zhoufeng Zhang, Junle Qu

**Affiliations:** 1 School of Optoelectronic Engineering, Xidian University, Xi’an 710071, China; 2 CAS Key Laboratory of Spectral Imaging Technology, Xi’an Institute of Optics and Precision Mechanics, Chinese Academy of Sciences, Xi’an 710119, China; 3 Sino-German College of Intelligent Manufacturing, Shenzhen Technology University, Shenzhen 518118, China; 4 College of Physics and Optoelectronic Engineering, Shenzhen University, Shenzhen 518060, China

**Keywords:** hyperspectral microscopic imaging technology, machine learning, skin cancer, cancer classification, staging identification

## Abstract

Skin cancer, a common type of cancer, is generally divided into basal cell carcinoma (BCC), squamous cell carcinoma (SCC) and malignant melanoma (MM). The incidence of skin cancer has continued to increase worldwide in recent years. Early detection can greatly reduce its morbidity and mortality. Hyperspectral microscopic imaging (HMI) technology can be used as a powerful tool for skin cancer diagnosis by reflecting the changes in the physical structure and microenvironment of the sample through the differences in the HMI data cube. Based on spectral data, this work studied the staging identification of SCC and the influence of the selected region of interest (ROI) on the staging results. In the SCC staging identification process, the optimal result corresponded to the standard normal variate transformation (SNV) for spectra preprocessing, the partial least squares (PLS) for dimensionality reduction, the hold-out method for dataset partition and the random forest (RF) model for staging identification, with the highest staging accuracy of 0.952 ± 0.014, and a kappa value of 0.928 ± 0.022. By comparing the staging results based on spectral characteristics from the nuclear compartments and peripheral regions, the spectral data of the nuclear compartments were found to contribute more to the accurate staging of SCC.

## 1. Introduction

Skin cancer is a relatively common type of cancer and is generally divided into three categories: basal cell carcinoma (BCC), squamous cell carcinoma (SCC) and malignant melanoma (MM). The incidence of skin cancer has continued to increase worldwide in recent years, with patients covering all age groups, especially those in middle and old age. According to the statistics of the International Agency for Research on Cancer (IARC), there were 1.518 million new skin cancer patients and 121,000 new deaths worldwide in 2020. Therefore, the diagnosis and treatment of skin cancer has become a major public health problem worldwide. The traditional diagnosis of skin cancer is carried out by shave biopsy, punch biopsy and excisional biopsy of the lesion areas. All three biopsy methods will cause damage to the patient; moreover, the biopsy results mainly depend on the clinical experience of the doctors, and there is the possibility of misdiagnosis or missed diagnosis [1]. Hyperspectral microscopic imaging (HMI) technology has been developed as a non-contact optical diagnostic method in recent years [2]. HMI combines hyperspectral imaging (HSI) technology with microscopy to provide both spectral information and image information of the tissue that is to be measured. The spectral data in the HMI cube reveal the internal microenvironment changes in the samples through parameters such as waveform and intensity, and the microscopic image data can intuitively reflect the sample’s differences in structure with high spatial resolution. At the same time, the combination of HMI and machine learning can assist doctors in diagnosis and greatly improve the efficiency and accuracy of the diagnosis, as well as having a wide field of application in the future [3,4,5,6,7].

In 2018, Nansen et al. [8] designed a cancer detection system that incorporated HSI and machine learning to distinguish the different types of cancers. Using principal component analysis (PCA) to find the features for artificial neural networks (ANNs) and support vector machines (SVMs), different cancer types could be distinguished with an overall accuracy of 87.4% using an ANN solution whereas the SVM accuracy ranged from 73–88.9%. In 2019, Chen et al. [9] used H&E to stain hepatic carcinoma tissues and obtained spectral–spatial data from their nuclei using hyperspectral microscopy. The transmission spectra of the nuclei were used to train an SVM model for cell classification. Their sensitivity and specificity in the identification of cancer cells could be increased to 99% and 98%, respectively. In 2020, Wang et al. [10] presented an automatic approach for the measurement of the superficial spreading depth of cutaneous melanomas based on microscopic hyperspectral imaging technology. An edge-detection method combined with kernel minimum noise fraction was used to extract the skin granular layer; the least squares SVM based on characteristic spectrum supervision was used to identify malignant melanocytes with an accuracy of more than 85%. Notarstefano et al. [11] analyzed tissue samples with diagnosis of pancreatic ductal adenocarcinoma and pancreatic neuroendocrine tumor using Fourier transform infrared HSI by means of both multivariate and univariate analyses and identified definite spectral markers of the different lesions. Their study showed that the malignant lesions were recognizable both from healthy/dysplastic pancreatic tissues and between each other. In 2021, Liu et al. [12] used an HMI system to identify 4’,6-diamidino-2-phenylindole (DAPI)-stained liver cancer cells. They took advantage of DAPI’s sensitivity to DNA, and used DAPI’s fluorescence intensity and spectral shape as features to identify liver cancer tissue and normal tissue. Using the SVM classification model, the sensitivity and specificity for the identification of 1000 liver cancer samples were 99.3% and 99.1%, respectively. In 2022, van Vliet-Pérez et al. [13] assessed the feasibility of near-infrared HSI for the detection of epithelial ovarian cancer in ex vivo tissue samples. Hyperspectral images with 25 spectral bands were acquired from the resected tissues in the wavelength range of 665–975 nm. A linear SVM was employed to classify healthy and tumorous tissue. The performance of the classification was evaluated by leave-one-out cross-validation. It was proved that tumorous tissue could be classified with a sensitivity of 0.81, a specificity of 0.70, an area under the curve (AUC) of 0.83, and Matthew’s correlation coefficient of 0.41. 

In this paper, HMI data cubes of BCC, MM and SCC were collected by an HMI system. Based on spectral data, the staging identification of SCC was realized, and the influence of the selection of spectral regions on staging results was studied.

## 2. Materials and Methods

### 2.1. Materials

The skin tissue samples used in our experiments were purchased from Xi’an Alenabio (sample no: SK801c; Xi’an, China) and ZhongkeGuanghua (Xi’an) Intelligent Biotechnology (sample no: K683501; Xi’an, China). In total, there were 34 cases of BCC, 63 cases of SCC and 39 cases of MM. For the SCC staging study, there were 13 cases of stage I, 37 cases of stage II and 13 cases of stage III.

### 2.2. HMI System

**Figure 1:**
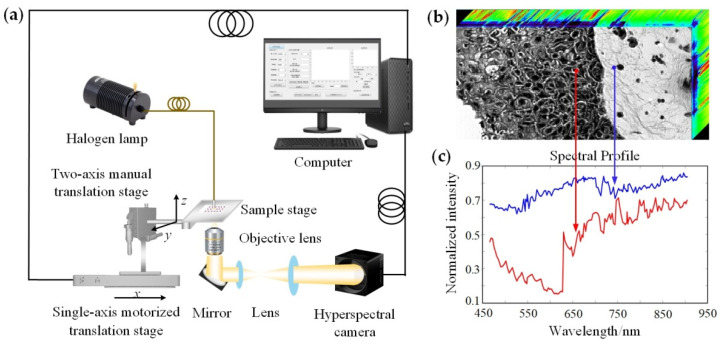
HMI system and data analysis. (**a**) Schematic diagram of the push-broom HMI system; (**b**) HMI data cube; (**c**) spectral profiles corresponding to the marked points in (**b**), the red and blue spectral curves corresponded to the red and blue points, respectively.

The schematic diagram of the push-broom HMI system used in our experiments is shown in Figure 1a. The main instruments included a tungsten halogen lamp light source (HL-2000, Ocean Insight; Dunedin, FL, USA), a hyperspectral camera (Xispec_MQ022HG-IM-LS150-VN2, XIMEA, Münster, Germany), an objective lens (10×/0.45, CFI Plan Apo Lambda, Nikon; Tokyo, Japan) and a motorized translation stage (OMS20-85, Sigma; Tokyo, Japan). The HMI system had a wavelength range of 465.5–905.1 nm, with a total of 151 bands and a spectral resolution of ~3 nm. The system magnification was 28.15×, the field of view was 400.18 μm × 192.47 μm, and the actual resolution was in the range of ~1.10–1.38 μm depending on the light wavelength. The light emitted from the tungsten halogen lamp illuminated the skin tissue on the sample stage. The transmitted light carrying the sample information was collected by the objective lens, and then passed through the mirror and lens group, and was directed to the hyperspectral camera. The motorized translation stage stepped in the x direction with a step size of 1 μm; thus, an HMI data cube containing image and spectral information with a size of 2048 × 985 × 151 was obtained, as shown in Figure 1b. Selecting the points or areas of interest for spectral analysis, the spectral profiles were displayed as in Figure 1c.

### 2.3. Methods

#### 2.3.1. Spectral Data Preprocessing

The spectral information of the HMI data cube can reflect the chemical composition and variation in the microenvironment within samples. In this paper, spectral data were used for SCC staging identification.

Generally, to reduce the influence of external noise and system scattering on the HMI system, spectral preprocessing is necessary [14,15], which can also improve the accuracy of subsequent procedures. The preprocessing methods used in this paper included the derivative method, standard normal variate (SNV) transformation and multiplicative scatter correction (MSC). The derivative method, such as the commonly used first derivative method (FD) and second derivative method (SD), can correct the spectral baseline and remove the background interference. The basic idea of SNV is to perform standard normal processing on the original spectrum, subtract the average value from the original spectrum, and then divide this by the standard deviation of the spectrum, so that the mean value of the spectrum is 0 and the standard deviation is 1. MSC first calculates the average spectrum, and then makes a univariate linear regression between each spectrum and the average spectrum. Both SNV and MSC can be used to eliminate the effect of scattering on the spectrum due to uneven particle distribution and particle size.

#### 2.3.2. Dimensionality Reduction

The HMI data cube contains high-dimensional information, which may result in an extremely complex computational procedure or even non-convergence and low accuracy in the classification model. Dimensionality reduction has been proven to be a powerful tool for high-dimensional data analysis because it can eliminate the redundances among data samples and simultaneously extract useful features. In this paper, two dimensionality reduction methods, namely, principal component analysis (PCA) and partial least squares (PLS), were used to reduce the dimensions of the HMI datasets to improve the accuracy of the model and speed up the algorithms [16,17]. PCA is a widely used linear unsupervised method, which can convert a set of observations of possibly correlated variables into as few uncorrelated variables, named principal components, as possible to retain the characteristics of the original data to the maximum extent; however, PCA cannot predict the dependent variables well. PLS is a linear, supervised, regression-based method that incorporates the ideas of principal component analysis and canonical correlation analysis. While used to reduce dimensionality, PLS can make the extracted feature variables not only generalize the information of the original variables well, but also have a strong explanatory power for the dependent variables, which is an improvement to the shortcomings of the PCA algorithm.

#### 2.3.3. Staging Identification Model

In the experiments, skin cancer staging identification models were established based on four classification methods: extreme learning machine (ELM), SVM, decision tree and random forest (RF) [18,19,20].

**Figure 2:**
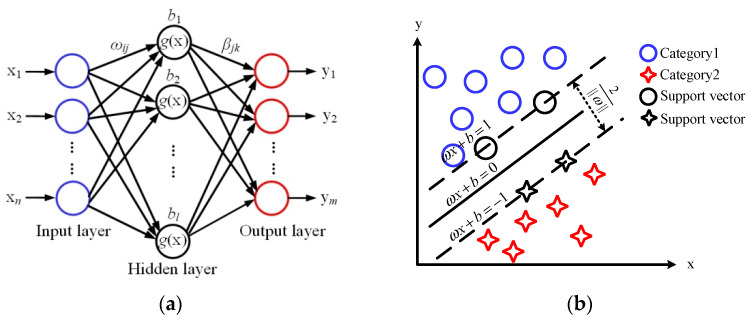

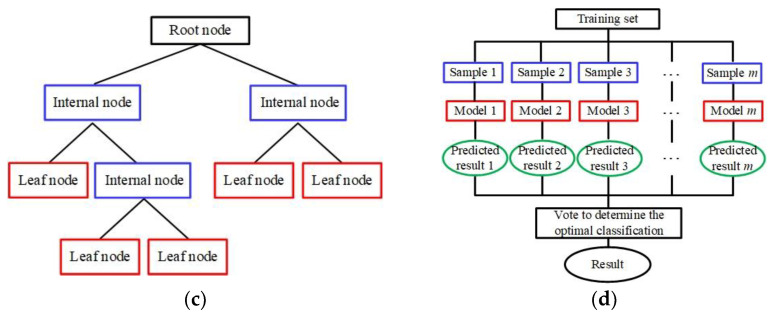
Classification models. (**a**) ELM; (**b**) SVM; (**c**) decision tree; (**d**) RF.

ELM is a single hidden-layer feedforward neural network, and its basic structure is shown in Figure 2a. The network consists of an input layer, output layer and hidden layer. *b* is the threshold of the hidden layer, and *l* is the number of neurons in the hidden layer. *ω_ij_* are the input weights connecting the input layer and hidden layer, and *β_jk_* are the output weights connecting the hidden layer and output layer. The input data can be converted into an interpretable output signal through the activation function *g*(x). In the implementation of the ELM algorithm, only the input parameter *l* is needed, *ω* and *b* are randomly generated after the input, and the output matrix of the hidden layer can be uniquely determined. In our experiments, the parameter *l* was set to 55.

The basic principle of the SVM algorithm is shown in Figure 2b. By solving the optimal hyperplane, the training dataset can be partitioned correctly and the geometric interval can be maximized. In the SVM algorithm, choosing the appropriate kernel function can effectively guarantee the classification accuracy. In this paper, the linear kernel function was selected after many repeated experiments.

A decision tree is a predictive model expressed in the form of a tree diagram, as shown in Figure 2c. A decision tree classifier includes root nodes, internal nodes and leaf nodes. In the process of generating a decision tree, three problems need to be solved: which feature to be chosen as the root node, which features to be chosen as the internal nodes, and when to stop splitting child nodes and achieve the stated goal.

RF uses the idea of ensemble learning to statistically analyze the voting results of multiple decision trees, and this process is shown in Figure 2d. In the RF classification algorithm, each decision tree selects some samples and some features. The number of decision trees determines the quality of the classification model. After repeated experiments, 15 decision trees were determined in the RF models for skin cancer staging identification.

## 3. Results

In this paper, the HMI system was used to collect the data cube of the skin cancer tissues. Then, SCC staging identification based on spectra was investigated.

### 3.1. SCC Staging Identification Based on Spectral Data

#### 3.1.1. Spectra Preprocessing

**Figure 3:**
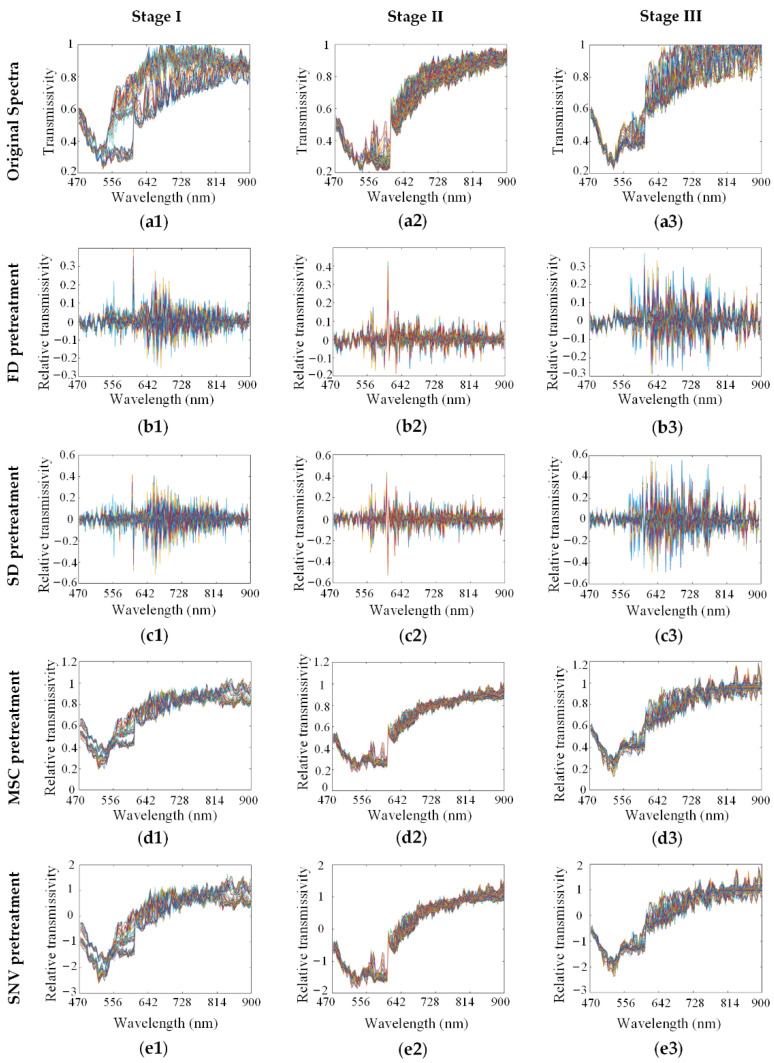
Spectra preprocessing of SCC at different clinical stages: stage I, stage II and stage III, respectively. (**a1**–**a3**) Original spectra; (**b1**–**b3**) preprocessed spectra by FD; (**c1**–**c3**) preprocessed spectra by SD; (**d1**–**d3**) preprocessed spectra by MSC; (**e1**–**e3**) preprocessed spectra by SNV. The different color lines represented the spectra of different sample pixels of the ROIs.

We used various methods to preprocess the spectra from the HMI data cube. As shown in Figure 3, the original spectra (a1–a3) of SCC at stage I, stage II and stage III in the SK801c tissue microarray were pretreated by FD (b1–b3), SD (c1–c3), MSC (d1–d3) and SNV (e1–e3), respectively. The effects of the preprocessing methods on modeling are discussed in the following process.

#### 3.1.2. Dimensionality Reduction

**Figure 4:**
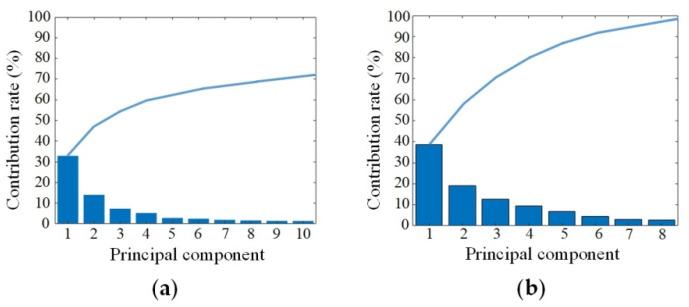
Principle component contribution rates with different dimensionality reduction methods. The columns represented the contribution rates of every principle component, and the lines represented the corresponding cumulative contribution rates. (**a**) PCA; (**b**) PLS.

PCA and PLS were performed on the original spectra of SCC at stages I, II and III to reduce the dimensions. Figure 4 shows the contribution of the extracted characteristic bands using PCA and PLS, respectively. The contribution rate of the first 10 principal components in PCA was 73%, and the contribution rate of the first 8 principal components in PLS was 96%. The results showed that the PLS method could more effectively extract the spectral features and greatly compress the HMI data cube. In this paper, the PLS was used for spectral dimensionality reduction, and the first 10 principal components were selected for SSC staging identification.

#### 3.1.3. SCC Staging Identification

The spectral data of SCC at stage I, II and III were preprocessed by FD, SD, MSC and SNV. Then, the preprocessed data were reduced dimensions with PLS for characteristic band extraction. Next, the dataset was divided into a training set and a test set with the ratio of 4:1 using the hold-out method. Finally, the SCC staging identification models of ELM, SVM, decision tree and RF were established. In this paper, accuracy and kappa values were used to evaluate the model performance. Overall accuracy is the most basic requirements in the classification, which is the number of correctly classified samples divided by the total number of classified samples. The kappa value is used to test the consistency of judgment and is obtained based on the confusion matrix. It ranges from 0 to 1, which can be divided into five groups to represent different levels of consistency: 0.0–0.20 for slight consistency, 0.20–0.40 for fair consistency, 0.40–0.60 for moderate consistency, 0.60–0.80 for substantial consistency and 0.80–1.0 for almost perfect consistency.

The staging results of each model are shown in Table 1. From the figures, it was clear that different spectra preprocessing methods had important impacts on the accuracy and kappa value of the staging models, and SNV performed best in each model. Among the four staging identification models, the RF model presented the optimum results with the highest accuracy and kappa value (0.952 ± 0.014, 0.928 ± 0.022), followed by ELM (0.948 ± 0.009, 0.922 ± 0.014), SVM (0.941 ± 0.015, 0.912 ± 0.022), and decision tree (0.885 ± 0.011, 0.827 ± 0.017).

### 3.2. Effects of Spectral ROIs on SCC Staging Identification

**Figure 5:**
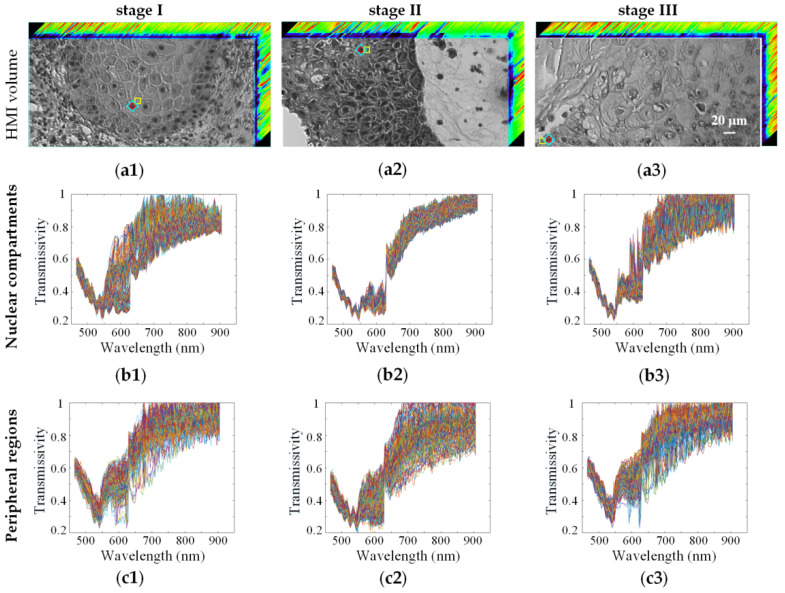
HMI images and original spectra of SCC at different clinical stages: stage I, stage II and stage III, respectively. (**a1**–**a3**) 3D HMI images. “◇”, “○” and “□” represented the selected spectral ROIs including the nucleus and surrounding areas, the nuclear compartments, and the peripheral regions, respectively; (**b1**–**b3**) original spectra of the nuclear compartments; (**c1**–**c3**) original spectra of the peripheral regions. The different color lines represented the spectra of different sample pixels of the ROIs.

Figure 5(a1–a3) show the HMI images of SCC at different clinical stages as an example. In the previous staging identification, larger ROIs including the nucleus and surrounding areas (for example, the regions marked with “◇”) were selected to obtain average spectra for post-processing. In this section, the spectral data of the nuclear compartments and the peripheral regions (for example, the regions marked with “○” and “□”, respectively) were analyzed respectively, as shown in Figure 5(b1–b3,c1–c3), to compare their effects on the staging results. According to the results listed in Table 1, SNV and MSC were selected for spectra preprocessing, and RF and ELM were selected as the staging models.

The SCC staging results based on spectral data from different ROIs are shown in Table 2. The findings indicated that the spectral ROIs influenced the staging results. When the selected spectral ROIs were the nuclear compartments, the staging accuracies were greatly improved: the highest accuracy reached 0.999 ± 0.001 and the kappa value was 0.998 ± 0.001. However, when the peripheral regions were selected as the spectral areas, the accuracies and kappa values were seriously reduced. Therefore, it could be concluded that the spectral data of the nuclear compartments contributed more than that of the peripheral regions to SCC staging.

In order to further verify the performance of the method discussed in this paper, 300 blind samples of SCC at stage I, stage II and stage III were used for staging identification. According to the above optimal results, the spectral data were from nuclear compartments, the spectra preprocessing method and the staging identification model were SNV and RF, respectively. Finally, the staging accuracy and kappa values of 0.807 ± 0.025 and 0.71 ± 0.018 were obtained, respectively. This result will provide reference for the staging of SCC.

## 4. Discussion and Conclusions

Skin cancer has attracted increasing attention, and its early diagnosis can help to improve the disease cure rates. In this paper, hyperspectral microscopic imaging (HMI) technology and machine learning are combined for skin cancer staging diagnosis. In the staging identification of SCC, spectral data were preprocessed by FD, SD, MSC and SNV; PCA and PLS were used to reduce the spectral dimensions; the hold-out method was used to divide the training set and test set; and the models of ELM, SVM, decision tree and RF were established for SCC staging identification. Moreover, the influence of the spectral ROIs on the staging results was also studied.

The results are as follows: SNV performed best in all of the spectral data preprocessing methods; PLS was better than PCA for dimensionality reduction; the RF model obtained the optimal SCC staging results with the highest staging accuracy and kappa value (0.952 ± 0.014, 0.928 ± 0.022); the spectral data of the nuclear compartments contributed more than that of the peripheral regions to SCC staging (accuracy and kappa value: (0.999 ± 0.001, 0.998 ± 0.001) vs. (0.609 ± 0.026, 0.414 ± 0.039)).

The results of this work show that the staging identification of SCC can be performed with high accuracy based on HMI technology and machine learning, which has great application potential in skin cancer diagnosis. This research can also provide a reference for the diagnosis of other diseases and lay a foundation for the study of spectral–spatial feature extraction for hyperspectral medical image classification.

## Figures and Tables

**Table 1:** SCC staging results based on spectral data.

Classification Model	Spectral Data Preprocessing	Accuracy	Kappa
ELM	FD	0.907 ± 0.015	0.861 ± 0.021
	SD	0.879 ± 0.024	0.818 ± 0.035
	MSC	0.937 ± 0.009	0.906 ± 0.014
	SNV	0.948 ± 0.009	0.922 ± 0.014
SVM	FD	0.907 ± 0.019	0.860 ± 0.029
	SD	0.841 ± 0.021	0.761 ± 0.032
	MSC	0.901 ± 0.031	0.861 ± 0.047
	SNV	0.941 ± 0.015	0.912 ± 0.022
Decision tree	FD	0.853 ± 0.032	0.779 ± 0.049
	SD	0.819 ± 0.015	0.729 ± 0.022
	MSC	0.866 ± 0.022	0.799 ± 0.034
	SNV	0.885 ± 0.011	0.827 ± 0.017
RF	FD	0.912 ± 0.019	0.868 ± 0.028
	SD	0.873 ± 0.029	0.809 ± 0.043
	MSC	0.942 ± 0.014	0.913 ± 0.020
	SNV	0.952 ± 0.014	0.928 ± 0.022

**Table 2:** SCC staging results based on spectral data from different ROIs.

Classification Model	Spectral Data Preprocessing	Nuclear Compartments		Peripheral Regions	
		Accuracy	Kappa	Accuracy	Kappa
ELM	MSC	0.996 ± 0.001	0.995 ± 0.001	0.599 ± 0.025	0.399 ± 0.037
	SNV	0.998 ± 0.002	0.997 ± 0.002	0.609 ± 0.026	0.414 ± 0.039
RF	MSC	0.998 ± 0.002	0.997 ± 0.003	0.541 ± 0.021	0.312 ± 0.032
	SNV	0.999 ± 0.001	0.998 ± 0.001	0.541 ± 0.019	0.312 ± 0.029

## Data Availability

Not applicable.
